# Environmental Conditions Associated with Four Index Cases of Pacific Oyster Mortality Syndrome (POMS) in *Crassostrea gigas* in Australia Between 2010 and 2024: Emergence or Introduction of *Ostreid herpesvirus-1*?

**DOI:** 10.3390/ani14213052

**Published:** 2024-10-22

**Authors:** Richard J. Whittington, Lachlan Ingram, Ana Rubio

**Affiliations:** 1 School of Veterinary Science, Faculty of Science, The University of Sydney, Camden, NSW 2570, Australia; 2 NSW Department of Primary Industries, Queanbeyan, NSW 2620, Australia; lachy.ingram@dpi.nsw.gov.au; 3 School of Life and Environmental Sciences, Faculty of Science, The University of Sydney, Camden, NSW 2570, Australia; 4 Environment Branch, Hornsby Shire Council, Hornsby, NSW 2077, Australia; arubio@hornsby.nsw.gov.au

**Keywords:** *Crassostrea gigas*, Pacific oyster mortality syndrome, OsHV-1, environmental conditions, climate change, disease emergence, aquaculture

## Abstract

**Simple Summary:**

Mass mortality of farmed Pacific oysters *Crassostrea gigas* caused by *Ostreid herpesvirus-1* is an international problem that has been linked to warm water temperatures. However, little is known about the environmental conditions leading up to the first appearance of the disease in a region—this is called an index case. Our aim was to look for consistent environmental features between the four index cases in Australia that occurred between 2010 and 2024. Water temperature alone did not explain these index cases but each one was preceded by unusually low rainfall and higher rates of temperature change that may be proxies for a range of undetermined factors. Comprehensive protocols for data acquisition are needed during future index cases.

**Abstract:**

Warm water temperature is a risk factor for recurrent mass mortality in farmed Pacific oysters *Crassostrea gigas* caused by *Ostreid herpesvirus-1*, but there is little information on environmental conditions when the disease first appears in a region—the index case. Environmental conditions between four index cases in Australia (2010, 2013, 2016 and 2024) were compared to provide insight into possible origins of the virus. Each index case was preceded by unusually low rainfall and higher rates of temperature change that could increase oyster susceptibility through thermal flux stress. Water temperature alone did not explain the index cases, there being no consistency in sea surface, estuary or air temperatures between them. Tidal cycles and chlorophyll-a levels were unremarkable, harmful algae were present in all index cases and anthropogenic environmental contamination was unlikely. The lack of an interpretable change in the estuarine environment suggests the recent introduction of OsHV-1; however, viral emergence from a local reservoir cannot be excluded. Future events will be difficult to predict. Temperature flux and rainfall are likely important, but they are proxies for a range of undetermined factors and to identify these, it will be necessary to develop comprehensive protocols for data acquisition during future index cases.

## 1. Introduction

The Pacific oyster (*Crassostrea gigas*) is an important species in aquaculture. Global production in 2021 was reported to be 626,000 tonnes valued at USD 1286 M [1], but these are underestimates due to aggregation of data for this species with cupped oysters which are the most important cultivated molluscs [2]. The tolerance of this species to a wide range of temperatures and salinity, and its rapid growth rate and ease of hatchery production have led to its introduction from northeast Asia into many countries for coastal aquaculture development [2]. There has been an increase in mass mortality events associated with the intensification of cultivation from the 1970s, and these are now a widespread international problem [3,4,5].

Mass mortality in *C. gigas* is associated with high water temperature, opportunistic pathogens, nutrition, reproduction and stress [6,7,8,9]. However, since 2008 *Ostreid herpesvirus-1* (OsHV-1) *uVar* [10] has been recognised as the main cause of mass mortality in Europe [11,12,13]. The condition is now known as Pacific oyster mortality syndrome (POMS) and cases continue to appear in new regions. Mitigation strategies have been devised including modification of husbandry techniques, breeding for disease resistance, diversification of farming to include other aquaculture species and vaccination [5,14,15].

**Figure 1:**
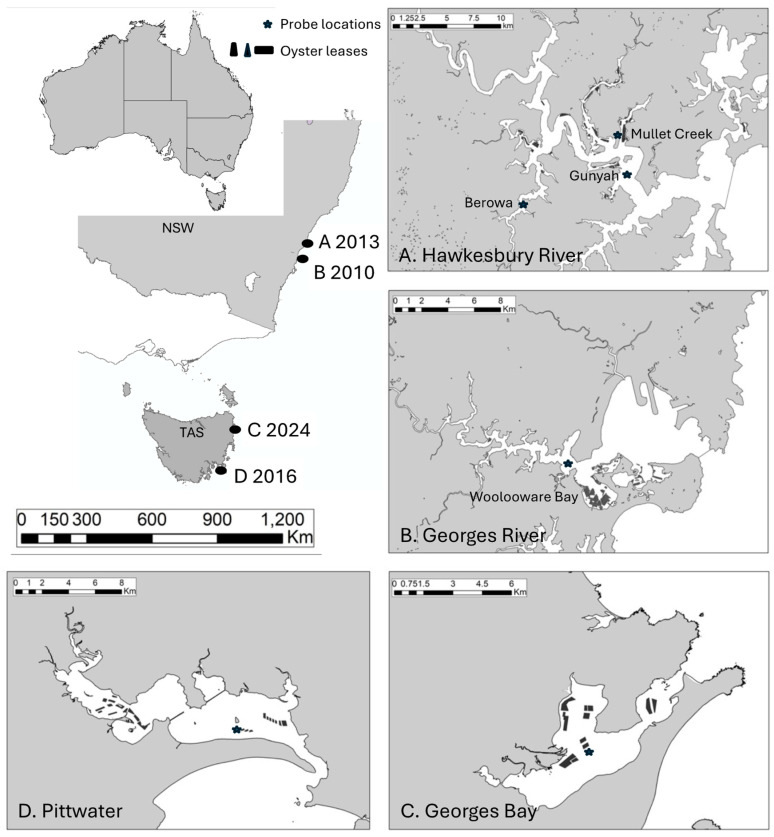
Location and dates of POMS index cases in Australia between 2010 and 2024, including the sites of the oyster leases and the real-time probe monitoring stations. NSW, New South Wales; TAS, Tasmania.

The first outbreak of POMS in Australia was detected in 2010 [16]. Outbreaks in new regions followed in 2013, 2016 and 2024 (Figure 1). 

These were the first known disease outbreaks in well-separated geographic areas and are termed index cases. Each outbreak had an immediate, significant, economic impact. Most of the farmed *C. gigas* population comprising millions of oysters died in the first three outbreaks [17,18] while 50–60% died in the fourth [19]. The origin of the virus is necessarily of considerable interest. It was assumed that it came from Europe where it was endemic, or from New Zealand where it was detected in 2010 [20], but it is now known that the Australian viruses are in a unique clade of OsHV-1 that is unknown from other parts of the world [21]. Furthermore, different variants of OsHV-1 were involved in three of the index cases [21]; the fourth has not yet been investigated. By inference, there were separate viral origins for these index cases in Australia. 

A new disease can appear when (i) an innocuous local virus mutates and becomes pathogenic; (ii) a pathogenic virus crosses into farmed oysters from wild reservoir hosts in which it may be innocuous; (iii) a pathogenic virus is moved by humans into a naïve host population that lacks immunity or (iv) a host population becomes immunologically compromised and more susceptible to a pathogen; this can be due to environmental stressors. These factors can occur in combination. The wild mollusc population is a potential reservoir for OsHV-1 because it can infect many species—with or without disease—and transmission can occur between different species [22,23,24,25]. In France, OsHV-1 *uVar* is thought to have replaced other strains of OsHV-1 from 2008, possibly reflecting the first or second mechanisms of disease occurrence [10]. Since then, OsHV-1 *uVar* has spread between countries in Europe through unrestricted movements of farmed oysters [13], illustrating the third mechanism, which is very common in aquaculture [26]. Husbandry conditions in intensive aquaculture contribute to environmental stressors as part of the fourth mechanism [27]. 

Confirming whether a pathogen has a local origin or has been introduced from elsewhere by humans is important in disease control because the prevention strategies for the two scenarios differ [28]. Human-assisted spread is often assumed; therefore, biosecurity measures such as animal movement restrictions are imposed that invariably disrupt farming businesses. In Australia, there is no evidence of human-assisted spread of OsHV-1. An important question is whether changes in the environment might have enabled local pathogen emergence. This could occur through any kind of habitat change that brings host species into proximity allowing pathogen exchange and new viral variants to emerge [29]. Investigation of environmental changes around the time of index cases may reveal indirect evidence for processes that could affect population dynamics of wild mollusc populations, *C. gigas* metabolism, stress and immunity, viral replication and bacterial populations. Bacterial proliferation causes death of individual oysters following viral-induced immunosuppression [30].

It is well known that when OsHV-1 is established in an estuary, seasonally recurrent POMS is associated with high water temperatures, and it follows that climate change and marine heatwaves may lead to a higher incidence of POMS, a feature confirmed by regression modelling [31]. However, there have been no studies investigating the role of the environment in the initial emergence of POMS. Did climate change play a role? If OsHV-1 acquired virulence through mutation and/or moved out of an environmental reservoir, was this facilitated by specific environmental conditions? If consistent environmental triggers for emergence were to be identified, is it possible to predict future emergence events and take steps to mitigate the losses? Furthermore, if environmental change triggers disease emergence, can biosecurity policies based on default assumptions of anthropogenic viral translocation be relaxed, to reduce costs to farmers?

**Figure 2:**
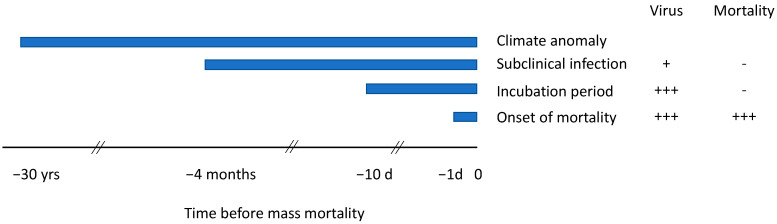
Periods for analysis with quantities of OsHV-1 and mortality observed in oysters.

POMS is preceded by an incubation period of 1 to 10 days during which OsHV-1 replicates to a high titre within oyster tissues and millions of virions are released into seawater before the oyster dies [32,33,34,35,36]. The rate is dose- and temperature-dependent so there can be exponential amplification under the right conditions [33,37,38]. Importantly, at the population level, the incubation period is preceded by subclinical infection during which a low concentration of the virus is present in some individual oysters in the absence of mortality. Subclinical infection lasting more than 3 months was observed in the Hawkesbury River New South Wales (NSW) index case through the use of sentinel oysters tested regularly for OsHV-1 in anticipation of an eventual outbreak of POMS [17]. Climate anomalies may favour viral emergence. This complex pathogenesis requires environmental conditions to be permissive at each of these stages, illustrated schematically in Figure 2.

Limited environmental data have been reported for the Georges and Hawkesbury River index cases, but none have been reported for the two events in Tasmania (TAS) [16,17,18]. The aim of this study was to investigate environmental parameters in more detail and to look for consistent features between the four index cases. Identification of the conditions permissive for viral emergence, subclinical infection, incubation of the virus and mass mortality may provide insights that could be used to predict future OsHV-1 index cases.

## 2. Materials and Methods

### 2.1. Study Design and Definitions

Retrospective analyses of environmental data were conducted to describe the conditions that coincided with the stages of the pathogenesis of POMS. 

An index case was defined as the first POMS event in a region. Index-case dates were based on notification of mass mortality by oyster farmers to government authorities (Table 1). The index case period was defined as the period commencing 24 h prior to 12:00 midday on the day of notification because farmers conducted routine observations of oysters in the morning. 

Consistent with the conventional understanding of the pathogenesis of infectious diseases and laboratory experiments on the transmission of OsHV-1 [33], mortality is preceded by exposure to the virus and an incubation period during which viral loads increase. The definition of incubation period was informed by prior daily observations in experimental infections and field trials in which the reported periods ranged from 1 to 10 days [32,33,34,35]. These values include the day of onset of mass mortality. In this paper, the incubation period was defined to commence 10 days before the onset of mass mortality (Table 1).

Subclinical infection was defined as the detection of OsHV-1 in oyster tissues, prior to the incubation period, in the absence of mass mortality, when followed by mass mortality. While subclinical infection is known to occur, its duration is difficult to define due to incomplete observations. Active surveillance of sentinel oysters in the Hawkesbury River revealed infection with OsHV-1 on 18 October 2012, more than 3 months before mass mortality occurred [17]. In Tasmania, opportunistic retrospective testing of archived samples collected from Great Oyster Bay Tasmania on 15 December 2015 revealed OsHV-1 approximately 1.5 months prior to the index case [39]. A subclinical infection period was assumed to precede each index case and was defined to begin four calendar months before the onset of mass mortality (Table 1). 

### 2.2. Disease Outbreaks and Locations

Four distinct mass mortality events form the basis for the present study (Table 1, Figure 1). (1) The first event was the index case for Australia: Oyster farmers at the Georges River estuary, NSW, notified government authorities of mass mortality on 24 November 2010 [16]. According to the diary records of the farmers, the spat were purchased from a hatchery in Tasmania and placed in the estuary on 16 November 2010. They were checked on 24 November when farmers noticed significant oyster mortalities. (2) The second index case began on 21 January 2013 when farmers at the Hawkesbury River NSW notified authorities of mass mortality [17]. (3) The third index case began three years later, on 27 January 2016 when farmers in TAS reported mass mortality affecting their oysters [18,40]. (4) The final index case commenced on 4 February 2024 with the report of mass mortalities in Georges Bay TAS [19].

The physical and biological attributes of these estuaries have been described in detail elsewhere [41,42,43,44]. The lower Georges River estuary, which includes Woolooware Bay, is an ocean embayment while the Hawkesbury River estuary is a drowned river valley; both have relatively unrestricted oceanic tidal flows. They are located 20 km south (−33.989011, 151.194878) and 50 km north (−33.564377, 151.278477) of Sydney Harbour, respectively (Figure 1). Most oyster cultivation in the Georges River occurs at the southern part of Botany Bay in Woolooware Bay within the lower estuary; the oyster leases are located in shallow areas among mudflats, surrounded by a foreshore dominated by mangroves and saltmarsh and in areas adjacent to natural remnant oyster reef. Oyster growing areas in the Hawkesbury River are located in each of the main creeks that branch off the main channel of the river in similar ecological, bathymetrical and geomorphological estuarine conditions to those in the Georges River. Lower Pittwater and Georges Bay are shallow coastal inlets and seawater inflows to each are constrained by a narrow inlet (Figure 1). 

### 2.3. Sources of Environmental Data

Local environmental data for the Georges River and the Hawkesbury River NSW were obtained from water quality monitoring programs run by the Sydney Catchment Management Authority and Hornsby Shire Council (HSC), respectively. Estuary water temperature (°C), salinity (ppt) and chlorophyll-a (Chl a) (µg/L) were measured at 15 min intervals using multi-sensor probes: Hydrolab DS5X sondes (Aqualab Scientific, Cromer, NSW, Australia) at Sylvania in the Georges River and YSI TM6820 sondes (John Morris Scientific, Chatswood, NSW, Australia) at Gunyah in the Hawkesbury River. These probes were deployed 1.0 m below the water surface suspended from a floating anchored buoy. Probes were calibrated and maintained to prevent marine fouling every three weeks in the warmer months. Readings from these probes were telemetered to a database. Due to concerns about the quality of the salinity data taken at the Georges River site, these data were not used in analyses but conditions at the time of the index case were known [16]. 

In Pittwater TAS, oyster lease water temperature was measured at 72 min intervals using Thermocron probes (OneTemp, Marleston, SA, Australia) deployed at a depth of 10–20 cm in floating baskets located on the farm at the site of the index case. Probes were manually downloaded to a computer. The data were curated by the authors. Oyster lease water temperatures for Georges Bay TAS were obtained from a probe managed by SenseRight, Cambridge, Tasmania for Oysters, Tasmania.

Tide level data for Fort Denison, Sydney, were obtained from the NSW Government, Manly Hydraulics Laboratory. Water levels at the specific oyster leases in the Georges and Hawkesbury Rivers were quantified based on continuous measurements using calibrated Hobo depth probes (OneTemp) for one month in 2015 and had equivalent tide levels and timing to Fort Denison. Tide level data for Hobart TAS were obtained from the Battery Point tsunami gauge managed by the Australian Government Bureau of Meteorology (BOM). Tide levels in Lower Pittwater were continuously monitored by the authors for one month in 2017; there was poor temporal correspondence with Battery Point data due to shallow water effects but similar compound tides and the same spring/neap tide cycles occurred; therefore, the data for Battery Point were used for Lower Pittwater. Tide levels for Spring Bay TAS were obtained from the BOM tide gauge at Spring Bay (Station 092133). BOM predicted tides for Georges Bay at St Helens had a minor temporal delay and lower daily minima and maxima with a daily range within 0.1 m; data from both sources were used in different analyses.

Air temperatures for the Georges River were obtained from The Bureau of Meteorology for Sydney Airport (Weather station 66037, period April 1939–May 2015), which is located on Botany Bay approximately 10 km from the Woolooware Bay oyster lease. Air temperatures measured every 15 min were obtained from the HSC Berowa-Calabash probe located approximately 10 km from the Mullet Creek oyster lease; air temperatures from this probe were shown to be highly correlated (R = 0.94) with those from an HSC probe installed subsequently at Mullet Creek. Air temperatures for Lower Pittwater and Georges Bay TAS were obtained from the BOM automated weather stations at Hobart Airport (Weather station 94250) 7 km away from the oyster farm and St Helens Aerodrome (Weather station 92120) 3.8 km away, respectively.

Long-term data for rainfall, minimum, maximum and average air temperature were obtained from the SILO database (https://www.longpaddock.qld.gov.au/silo/) (accessed 1 July 2024) which provides daily (1889—current day) data for a range of climatological variables on a 5 × 5 km grid for the Australian continent [45]. The data are obtained primarily (>80%) from local weather stations within the 5 × 5 km grid, but in instances where data are missing (non-recorded, instrument failure), they are interpolated from surrounding stations. Data for the Georges River were obtained from the grid centred at latitude −34.00, longitude 151.15; those for the Hawkesbury River at −33.55, 151.25; those for Lower Pittwater at −42.80, 147.60; and those for Georges Bay at −41.30, 148.30.

Daily oceanic sea surface temperatures (SST) and SST anomaly data were obtained from the US National Oceanic and Atmospheric Administration (http://www.esrl.noaa.gov/psd/data/gridded/data.noaa.oisst.v2.highres.html) (accessed 1 July 2024) for −34.125, 151.125 (Georges River/Botany Bay mouth, NSW), −33.625, 151.375 (Hawkesbury River mouth, NSW), −43.125, 147.625 (Storm Bay, Tasmania) and −41.265, 148.355 (Georges Bay mouth, Tasmania) for the period 1 September 1981 to 3 February 2024.

“Snapshot” sea surface temperature images of south-eastern Australia for the index case dates were downloaded from https://oceancurrent.aodn.org.au (accessed 1 July 2024) and comprise data from a polar-orbiting satellite that measures SST in cloud-free regions overlaid on prior quality data.

Limited data on harmful phytoplankton were available from monitoring conducted approximately monthly by (i) the NSW Food Authority as part of the Shellfish Quality Assurance Program [46] and the Hornsby Shire Council water quality monitoring program and (ii) the Shellfish Market Access Program (ShellMAP) administered by the Department of Natural Resources and Environment, Government of Tasmania (https://nre.tas.gov.au/aquaculture/shellmap) (accessed 30 September 2024). Results for harmful algal species with the potential to adversely affect *C. gigas* [47] were extracted from larger datasets. 

### 2.4. Data Analysis

Time-series plots were used to summarise environmental conditions during each mass mortality event, the preceding incubation period and the subclinical infection period, using dates defined in Table 1 and illustrated in Figure 2. Data at 15 min intervals were used to calculate parameters for the 10 days prior to the onset of mass mortality while daily means were used for the subclinical infection period.

All data analysis was undertaken using R (R Core Team, 2023, Vienna, Austria). The various data sources (tidal data, water quality variables, air and water temperature) were first checked to ensure that no obvious outliers or other anomalous data were present. Where data were collected at larger time intervals (either 30 or 60 min) than 15 min (the smallest interval), the intervening 15 min intervals were filled using a linear regression interpolation using imputeTS [48]. In the case of the Georges River dataset, some estuary water temperature and Chl a data were missing for the period 23 July 2010 to 31 July 2010 inclusive, in which case we used missRanger [49] to apply a random forest machine learning algorithm to gap-fill data. The use of this package allowed for non-linear relationships to be incorporated into the interpolation process. Data used to gap fill the missing estuary water temperature and Chl a data include air temperature, water temperature, rainfall, turbidity, tide height, electrical conductivity, dissolved oxygen and sea surface temperature from both the site with missing data as well as a nearby upstream site. The out-of-bag error for estuary water temperature was 0.0001 and for Chl a was 0.0279.

In order to explore the role that water temperature may have played in driving the onset of index mortality, the package heatwaveR [50] was used to investigate periods of time when water temperature exceeded 15, 16, 17, 18, 19, 20, 21, 22, 23, 24 and 25 °C for a continuous period of 3 h during the incubation period for each of the index sites. For this analysis, the 15 min data were used. To examine longer-term changes in water temperature during the subclinical period (4 months), the 15 min data were aggregated into a daily average temperature and then the number of events that exceeded the temperatures for three or more days was determined.

Spearman correlation analysis was undertaken of daily mean estuary temperatures and SST for each site (GR, 184 days; HWY, 764 days; LP, 153 days; GB, 125 days).

In order to calculate anomalies with respect to long-term climatological data, daily data of minimum, mean and maximum air temperatures and rainfall from 1 September, 1981, were downloaded from SILO for the grid location of each site; this period matched available SST anomaly data. For each index case, a 7-day centred rolling mean for the period was calculated and then a mean each day of the year was calculated and subtracted from each date. For rainfall data, the daily rainfall was summed to provide the total rainfall for each month. The mean of monthly rainfall was determined and then subtracted from each month of rainfall.

## 3. Results

The dates defined for the onset of mass mortality, the incubation period and the subclinical infection period for each index case location are shown in Table 1.

### 3.1. Environmental Conditions for the Day of Onset of Mass Mortality

**Figure 3:**
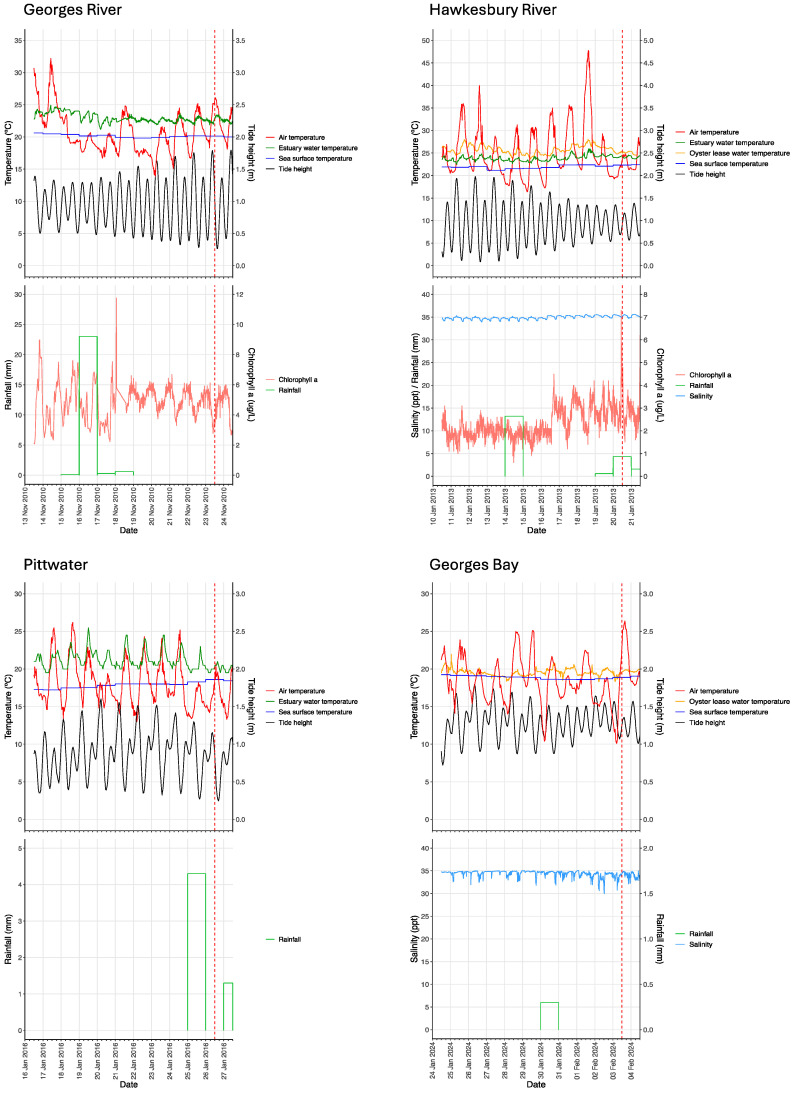
Index case and incubation period conditions. Values are data recorded at 15 min intervals over the 10 days prior to and including the onset of mass mortality. Dashed vertical line—onset of index case.

Local environmental conditions for 24 h, ending at midday, on the index case day, are presented in Table 2 and Figure 3. 

Mean daily air temperature was similar for Georges River NSW, Hawkesbury River NSW and Georges Bay TAS (21.3 to 23.4 °C) but it was much cooler for Lower Pittwater TAS (16.8 °C). The daily temperature range was similar for all four locations, being 7.2 to 10.6 °C.

The sea surface temperature at the mouth of each estuary was about 2 °C cooler in the Georges River (20.1 °C) than in the Hawkesbury River (22.4 °C) and both were higher than the two Tasmanian locations (18.5 to 19.0 °C). Mean daily estuary/oyster-lease water temperatures were up to 2.6 °C warmer than coastal sea surface water temperatures.

**Figure 4:**
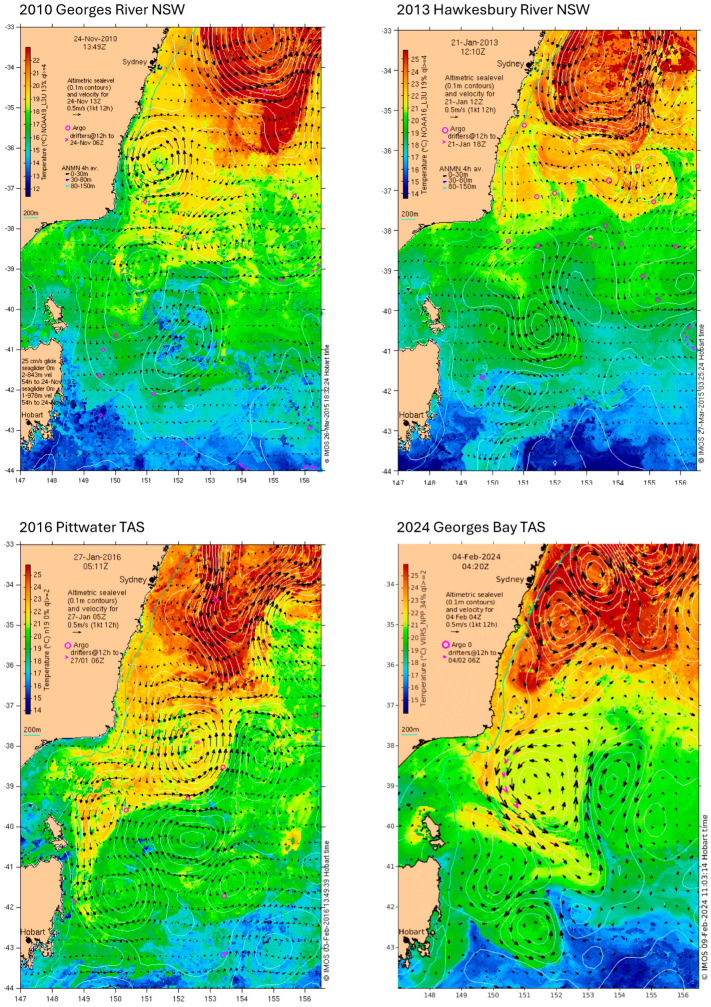
Snapshot sea surface temperature images for south-eastern Australia for each index case. Reproduced with acknowledgement to Australia’s Integrated Marine Observing System (IMOS).

Snapshot sea surface temperatures for each location are shown in Figure 4. At the time of each index case in NSW, there was a warm tail of the south-flowing East Australia current lying offshore Sydney. The temperature of these currents was 23–24 °C on 16 November 2010 and 26–27 °C on 21 January 2013. The southward current travelled closer to the coast until around 500 km north of the Hawkesbury River mouth, and then, separated as it approached the Hawkesbury latitude. Therefore, towards the coastal edge, there was a gradual reduction in temperature of 3 °C for the Georges River 2010 index case and 6 °C for the Hawkesbury River 2013 index case. In January 2016, the East Australia current penetrated much further south; eddies from it reached Bass Strait at 21 °C to 22 °C and flowed down the east coast of Tasmania. However, a band of cooler water 18 °C to 19 °C separated the warmer water from the coast. In February 2024, the East Australia current did not influence the east coast of Tasmania where water temperatures were 18 °C to 19 °C (Figure 4).

Estuary salinity levels during the index case were a little lower in the Georges River NSW (31 ppt) [16] than in the Hawkesbury River NSW (35 ppt) and Georges Bay TAS (34 ppt). They were not directly measured in Lower Pittwater TAS but can be assumed to have been high (oceanic) due to low rainfall in the region (see Table 2 and Table 3, Figure 3 and discussion).

**Figure 5:**
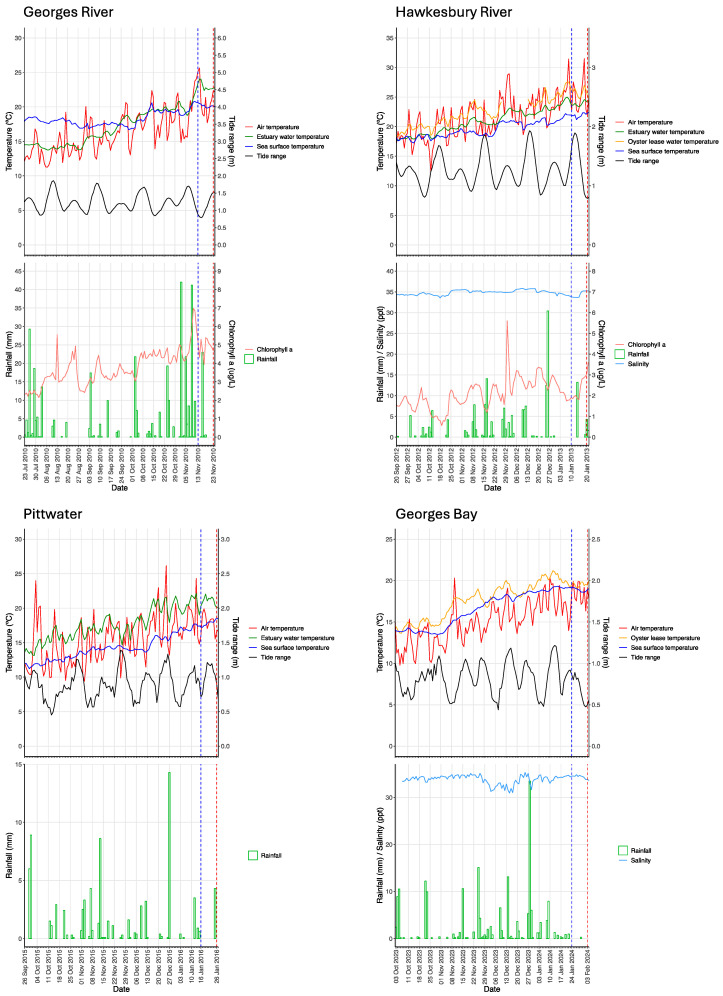
Subclinical infection period conditions. Data are daily averages of data collected at 15 min intervals and the daily tidal amplitude. Dashed blue vertical line—start of incubation period; dashed red vertical line—onset of mass mortality.

The index cases were not associated with any particular level of tide: in the Georges River NSW, the index case coincided with spring tides representing large differences between high and low water levels; in the Hawkesbury River NSW and Georges Bay TAS, it coincided with neap tides when there were relatively small differences between high and low water levels; in Lower Pittwater TAS, it occurred approaching neap tides (Table 2). The absolute water levels are shown in Figure 3 while the daily ranges of water level, which make it easier to discern spring and neap tides, are illustrated in Figure 5. 

Estuary chlorophyll-a levels were low in both index cases in NSW (<5 µg/L) but were not available for either location in TAS. 

### 3.2. Environmental Conditions for the 10-Day Incubation Period Leading to Mass Mortality

The environmental conditions during the 10-day incubation period prior to the onset of mass mortality are presented in Table 3 and Figure 3.

The mean air temperatures differed between locations, being warmest in the Hawkesbury River (25 °C) and coolest in Tasmania (18 °C to 19 °C). The highest daily maximum occurred during the Hawkesbury River incubation period in January 2013 (47.8 °C) (Table 3). Daily maximum air temperature exceeded 35 °C on 5 of 10 days during the Hawkesbury River incubation period, sometimes coinciding with low tide when oyster baskets potentially had emerged from the water (Figure 3) and were exposed to extreme heat. The maximum air temperatures during the Georges River NSW and the two Tasmanian incubation periods were 32 °C and 26 °C to 28 °C, respectively (Table 3).

Mean sea surface temperatures at the mouth of the estuary over the 10-day incubation periods were 2 °C warmer for the Hawkesbury River NSW (22 °C) than for the Georges River NSW (20 °C) and both of these were higher than both Tasmanian index cases (18 °C to 19 °C). 

Within the estuary/oyster-lease, mean water temperature during the incubation period approached or exceeded 20 °C at each location and was highest in the Hawkesbury River (23.9 °C estuary, 25.8 °C oyster-lease). The maximum water temperatures recorded at each location ranged from 22.0 °C in Georges Bay TAS to 28.1 °C in the Hawkesbury River NSW, while the water temperature range over 10 days was greater for Lower Pittwater (6.0 °C) compared to the other locations (3.7 °C to 4.5 °C) (Table 3). The oyster-lease water temperature had a cyclical pattern coincident with tides, being approximately 2 °C warmer during daytime low tides (Figure 3). The coastal sea surface temperature at the mouth of each estuary was about 1–2 °C cooler than the corresponding estuary temperature, and an upstream gradient of increasing water temperature was observed in the Hawkesbury River, the oyster-lease being 2 °C to 4 °C warmer than the estuary (Table 3, Figure 3).

The total durations of exposure to different threshold water temperatures for periods greater than 3 h during the 10-day incubation period are shown in Table 4. In three index cases, oysters were exposed to water temperatures exceeding 22 °C and averaging at least 0.7 °C higher than this, but for variable durations (42 h to 240 h between locations) before the onset of mass mortality. In Georges Bay TAS, there was less cumulative thermal exposure, with water temperatures exceeding 20 °C for a cumulative duration of only 24 h.

Reliable salinity measurements were not available for the Georges River NSW during the incubation period but were assumed to be a little lower than those during the Hawkesbury River NSW incubation period (34.3 ppt), based on one reading (31 ppt) reported for the end of the period [16]. There was 24 mm of rainfall during the incubation period, which would explain the relatively low salinity value for the Georges River (Table 3). The Hawkesbury River probe location was in the main channel of the estuary where there was little dilution effect from 16 mm local rainfall. Salinity data were not available for Lower Pittwater TAS, but levels were assumed to be oceanic due to low rainfall in the region during the incubation period (total 4 mm) (see Table 3, Figure 3 and discussion). There was >30 ppt salinity throughout the incubation period in Georges Bay TAS (Table 3, Figure 3).

The mean estuary chlorophyll-a level was 4.9 µg/L in the Georges River (Table 3). However, rainfall run-off in the Georges River catchment resulted in an increase in chlorophyll-a levels to about 12 µg/L one week preceding the outbreak (Figure 3). In the Hawkesbury River, mean estuary chlorophyll-a levels were 2.3 µg/L across the 10-day incubation period; however, there was a systematic shift in chlorophyll-a level, which approximately doubled about halfway through the incubation period (Figure 3). Chlorophyll-a data were not available for either location in TAS.

The incubation periods at each location included both spring and neap tides; the former occurring at the end of the incubation period in the Georges River NSW and the latter at the end of the incubation periods in the Hawkesbury River NSW and both locations in TAS. The absolute water levels are shown in Figure 3, while the daily ranges of water levels, which make it easier to discern spring and neap tides, are illustrated in Figure 5. 

### 3.3. Environmental Conditions for the 4-Month Subclinical Infection Period

Environmental conditions during the 4-month subclinical infection period are shown in Table 5 and Figure 5. 

Over this long period, there were seasonal increases in air and water temperatures. Mean daily air temperatures for the Georges River NSW increased from about 11 °C to 26 °C while those for the Hawkesbury River NSW increased from 12 °C to 32 °C. In TAS, mean daily air temperatures increased from approximately 9 °C to 21 °C for Georges Bay and to 26 °C for Lower Pittwater (Figure 5).

Sea surface water temperatures at the mouth of the Georges River NSW estuary in summer 2010–2011 were slightly higher compared to the Hawkesbury River NSW in summer 2012–2013. At the Georges River mouth, six weeks prior to the outbreak, SST increased from about 17 °C in two incremental steps of 2 °C (Figure 5). The first increment was from 18 °C to 20 °C in 4 days and the second increment twenty days later was from 18.7 °C to 20.8 °C in 6 days. At the Hawkesbury River estuary mouth, SST increased in 2 °C increments over periods of 4 to 6 days (Figure 5). In TAS, the SST increased by 3 °C to 16 °C from about 6 weeks prior to the index case then progressively climbed to a maximum of 18.6 °C at Lower Pittwater and 19.3 °C at the mouth of Georges Bay (Figure 5, Table 5).

Sea surface temperature was correlated with the mean daily estuary water temperature at each location (Georges River NSW R = 0.75, Hawkesbury River NSW 0.77, Lower Pittwater TAS 0.93, Georges Bay TAS 0.92). 

In the Georges River NSW, the average daily estuary water temperature progressively increased from 13.8 to 24.1 °C during this period. In the Hawkesbury River NSW, it rose from approximately 18 °C, peaking in January 2013 at 25 °C (Figure 5). Water temperatures at the oyster leases in the bay were 2 to 4 °C warmer than the estuary water temperatures, the greatest differences occurring on days when air temperature was highest (Figure 5). The average daily estuary and oyster-lease water temperatures on 18 October 2012, when subclinical infection was first detected in *C. gigas*, in the Hawkesbury River were 19.3 °C and 21.1 °C, respectively (Figure 5). At both locations in TAS, the mean daily oyster-lease water temperature progressively increased from about 13–14 °C to 21–22 °C by the end of the subclinical infection period.

Oysters were exposed to mean daily water temperatures exceeding 20 °C for cumulative durations of 15 to 31 days during the 4-month subclinical infection period in three of the index cases but for much longer in the Hawkesbury River NSW (81 days) (Table 6). The rate of increase in water temperature to this threshold was highly variable, ranging from 0.1 °C/day to 0.6 °C/day depending on location. The rates of increase in water temperature tended to be higher at the higher thresholds at each location (Table 6). For example, in the Georges River, the rate of increase in water temperature above the threshold of 15 degrees was 0.1 °C/day from 84 days before the onset of mass mortality, but within 18 days of the onset, it increased above 20 °C at a rate of 0.5 °C/day. Rates of increase in Georges Bay TAS were lower than for the other locations, with the highest (0.3 °C/day) occurring above a threshold of 20 °C. 

**Figure 6:**
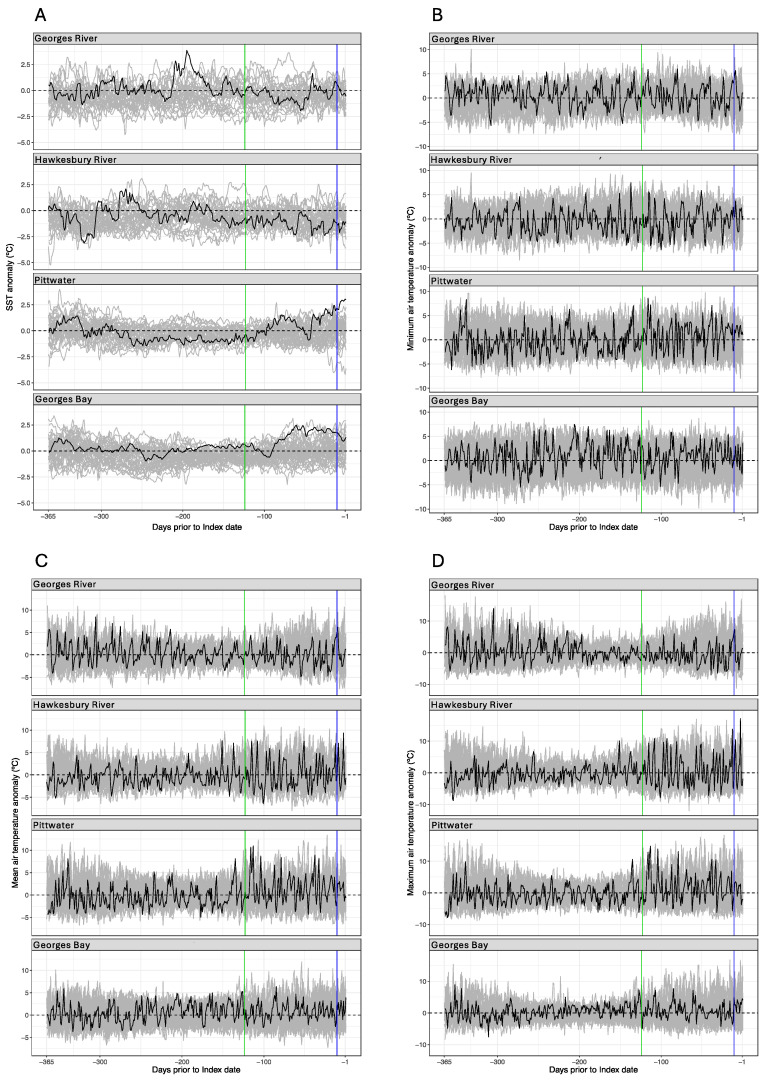
Daily temperature anomalies for the year prior to each index case, with reference to the period from 1 September, 1981: Panel (**A**), sea surface temperature; Panel (**B**), minimum air temperature; Panel (**C**), mean air temperature; Panel (**D**), maximum air temperature. Black line, daily anomaly for the year immediately prior to the index case that ended at day 0; grey line, daily anomaly for the reference period; vertical green line, start of subclinical infection period; vertical blue line, start of incubation period.

Salinity measurements were not available for the Georges River NSW, but there were numerous rain events during the subclinical infection period totalling 357 mm of rainfall (Figure 5). Therefore, it can be assumed that salinity fluctuated. In the Hawkesbury River, salinity levels were relatively stable due to the absence of heavy local or regional rainfall (total 174 mm during the subclinical infection period) and consequently, there was not an excess of freshwater inflow into the estuary. Mean estuary salinity levels increased slightly from approximately 34 ppt in September 2012 to 35–36 ppt in December 2012 (Figure 5). The average daily estuary salinity level on 18/10/12, when subclinical infection was first detected in *C. gigas,* was 33.6 ppt (Figure 5). Salinity measurements were not available for Lower Pittwater TAS but were assumed to be high (oceanic) due to low rainfall in the region (total 82 mm) and the oceanic characteristic of this estuary (Table 5, Figure 5, Figure 6B, see discussion). Salinity levels fluctuated but were mostly above 32 ppt in Georges Bay TAS throughout the 4-month subclinical infection period, averaging 33 ppt (Table 5, Figure 5).

In the Georges River NSW, chlorophyll-a levels were below 5 ug/L during the months prior to the outbreak, but gradually increased to around 5 µg/L in the weeks prior to the outbreak (Figure 5). In the Hawkesbury River NSW, mean estuary chlorophyll-a levels were relatively low and stable throughout most of the incubation period, not exceeding 5.6 µg/L (Figure 5). Chlorophyll-a data were not available for the two locations in TAS.

The first detection of subclinical infection in the Hawkesbury River on 18 October 2012 coincided with spring tides (Figure 5) as did the first detection of OsHV-1 in TAS (Figure 5). There were no data for the Georges River NSW or Georges Bay TAS prior to the index case because relevant tests for OsHV-1 were not performed for those locations.

### 3.4. Sea Surface Temperature and Climate Anomalies

In the year prior to each index case, there were inconsistent sea surface temperature and atmospheric temperature patterns and there were no consistent anomalies between index cases that could be used to explain viral emergence (Figure 6). 

Similarly, there were no consistent sea surface temperature features that could explain the exit from the subclinical infection period into the incubation period. The mean daily sea surface temperature for the Georges River estuary was close to the long-term term average for much of the subclinical, incubation and index case periods. There was a different pattern for the Hawkesbury River where it was approximately 1 °C below at the onset of the subclinical period and approximately 2 °C below for the incubation and index case periods. In Lower Pittwater TAS, the mean daily sea surface temperature was approximately 1 °C below the long-term average at the onset of the subclinical period and approximately 2.5 °C above for the incubation and index case periods (Figure 6). It was also generally above the long-term average for Georges Bay TAS. However, with the exception of the 10-day incubation period for Lower Pittwater TAS, during which water temperatures were exceptionally high, there was no consistent anomaly (Figure 6).

The minimum, maximum and mean daily air temperatures were generally within long-term ranges at all four locations (Figure 6). However, a heatwave occurred in the Hawkesbury River region on 19 Jan 2013, two days prior to the index case, with air temperature ≥46 °C; these were the highest temperatures ever recorded in the Sydney area (Figure 6, Panel D). While oysters grown in intertidal cultivation structures are exposed to air at low tide, the spat that died in the Hawkesbury River index case were in subtidal baskets (always immersed) and, when the mortality event began, the extremely high air temperature coincided with high tide (Figure 3). One week earlier, during the incubation period, air temperatures of 35 to 40 °C did coincide with low tides, and there were obvious fluctuations in water temperature in each 24 h period due to the high air temperatures (Figure 3). 

**Figure 7:**
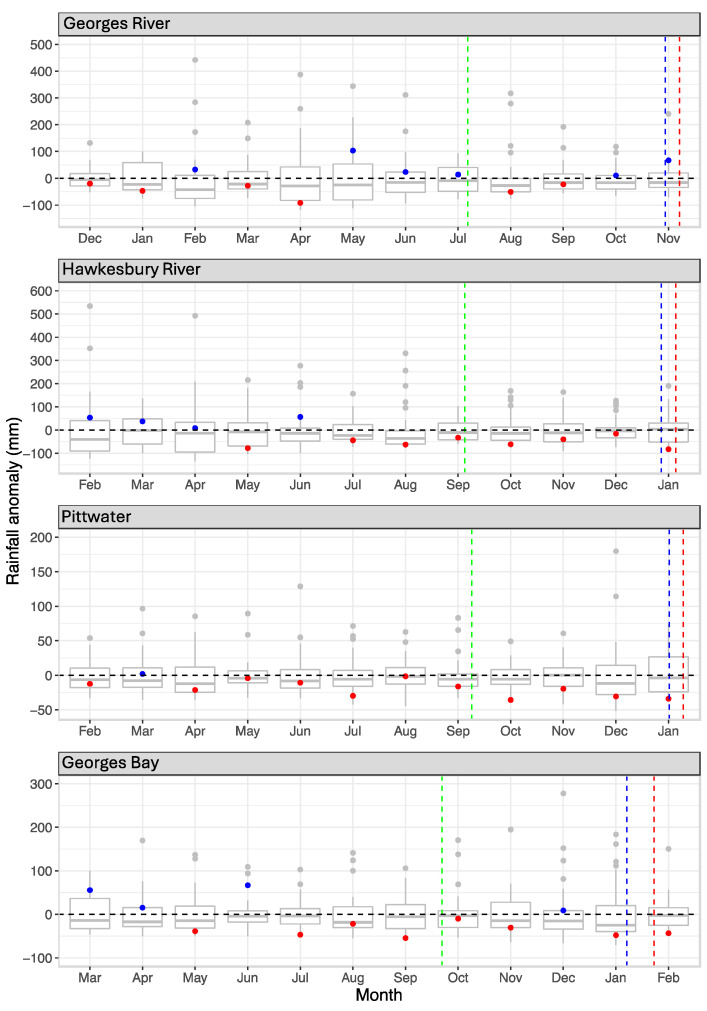
Monthly rainfall anomalies for the period 1 September 1981, to the Index case date for each site. The box shows the middle 50% of the data (interquartile range for 25 to 75 percentile); the median anomaly value is indicated by the line within the box; the whiskers show the range of the top and bottom 25% of values. Grey dots indicate outlier values. Blue (wetter) and red (drier) dots show anomaly values each month for the 12 months prior to each index case. Dashed green line, start of subclinical infection period; dashed blue line, start of incubation period; dashed red line, onset of mass mortality.

There was below-average rainfall for at least four months before the subclinical infection period and for at least 2 months within this period in all four index cases (Figure 7). Low monthly rainfall anomalies also occurred in the incubation period of three index cases (Figure 7). There was a high rainfall anomaly in the month of the incubation period in the Georges River NSW index case, but this was preceded by two dry months in the subclinical infection period.

### 3.5. Harmful Algae

Monitoring for harmful algae was conducted intermittently in each estuary. *Alexandrium catenella* was detected during the subclinical infection period close to oyster leases in the Georges River NSW in September (trace levels) and October 2010 (130 cells/L) and again between 12 and 9 November 2010 (1100 to 22,000 cells/L) during the incubation period. *A. catenella* was also present during the incubation period in the Hawkesbury River NSW close to the Gunyah water quality monitoring station in September 2012 (50 cells/L), on 24 October 2012 (300 cells/L) and 19 December 2012 (40 cells/L) but was not detected during the incubation period in January 2013.

During the subclinical infection period in Pittwater, *Dinophysis acuminata* was detected at 98 cells/L on 15 December 2015 and the *Pseudo-nitschia seriata* group at 2400 cell/L on 29 December 2015 while the latter was detected at 970 cells/L on 21 January 2016 during the incubation period.

A wider range of harmful algal species was detected in Georges Bay during the subclinical infection period: *Alexandrium* sp. 46 cells/L on 10 January 2024; *Cerataulina pelagica* 2850 cells/L on 29 November 2023 and 3800 cells/L on 10 January 2024; *Dinophysis acuminata* 47 cells/L on 29 November 2023; *Dinophysis acuta* 46 cells/L on 29 November 2023 and 10 January 2024; *Gymnodinium* sp 1900 cells/L on 30 October 2023 and 29 November 2023; the *Pseudo-nitschia delicatissima* group 1800 cells/L on 30 October 2023, 50,650 cells/L on 29 November 2023 and 46 cells/L on 10 January 2024; and the *Pseudo-nitschia seriata* group 25,000 cells/L on 30 October 2023, 14,200 cells/L on 29 November 2023 and 8000 cells/L on 10 January 2024.

## 4. Discussion

In this study, we looked for consistent environmental features between four index cases of POMS spaced over 14 years in an attempt to explain the appearance of OsHV-1 in *C. gigas*. After OsHV-1 was detected during the first index case in 2010, the other three locations were tested intensively in 2011 with negative results [51]; therefore, viral emergence or introduction occurred in those estuaries after 2011. We examined the environmental conditions leading up to actual or assumed subclinical infection periods that could explain the local emergence of the virus. We also examined the subclinical infection period to evaluate factors that could be involved in the progression from subclinical infection into the incubation period, as well as the incubation period and onset of mass mortality. Each of the environmental parameters for which there were data will be discussed in turn.

### 4.1. Temperature

Endemic POMS occurs only during the warmer months across Europe, the USA and Australasia [20,30,52,53,54,55,56,57,58,59], but the mass mortalities during these events can occur over a wide range of water temperatures. For example, in France, the range is 16 to 24 °C [60]. In a large study at 13 sites over a 600 km latitudinal gradient in France, OsHV-1 was detected and mortality began at each site when the water temperature reached 16 °C, reinforcing the results of an earlier study at fewer sites [53,61]. Rates of change in temperature, daily variability of temperature [53,57,61,62] and duration of exposure to elevated water temperatures [56] could also be important in pathogenesis together with factors correlated with temperature. These studies confirm the concept of a temperature threshold for mass mortality, but the threshold is several degrees higher in Australia than in Europe, both in the field and in laboratory challenge experiments [37], suggesting that other factors contribute to the disease.

Water temperature data were reported for the Georges River [16] and the Hawkesbury River index cases [17], but detailed analysis revealed substantial divergence in water temperature patterns between these and the other two index cases. When the mass mortalities began, ranked mean daily estuary/oyster-lease water temperature was 19.7, 20.1 °C, 22.7 °C and 25.1 °C, for Georges Bay TAS, Lower Pittwater TAS, Georges River NSW and Hawkesbury River NSW, respectively, a range of 5.4 °C. Based on experimental infections, it is known that a temperature above 20 °C is required for significant mortality due to the Australian variants of OsHV-1 [37]. During the 10-day incubation period, there was a wide divergence in the cumulative exposure time to water temperatures exceeding this threshold: 24 h to 240 h between locations. There was a similar divergence during the subclinical infection period with much longer exposure to relatively warm temperatures >20 °C in the Hawkesbury River (81 days) compared to the other locations (15 to 31 days). In all four index cases, the durations of exposure to water temperatures known to be permissive for mass mortality [37] were exceeded weeks before mass mortality began. OsHV-1 was present as a subclinical infection in *C. gigas* populations in Tasmania at least 43 days before disease onset in Lower Pittwater and in the Hawkesbury River NSW population 95 days prior to mass mortality [17,39]. Therefore, if temperature per se is the only factor for the emergence of OsHV-1 or progression from subclinical infection into an incubation period, then mass mortality in each index case would have commenced far sooner than was observed. That mass mortality did not occur sooner at each location is strong evidence that the virus was unable to transmit efficiently between oysters during the subclinical infection period and that other factors are required. The concept of subclinical OsHV-1 infection in POMS is seldom discussed, but it also occurs in endemically infected oyster populations [54,63,64,65].

One consistent feature between index cases was that the rates of increase in water temperature were higher as temperatures increased, even though these rates were lower overall in the Hawkesbury River NSW and Georges Bay TAS (Table 6). Rapidly changing temperatures would require rapid metabolic adaptation by oysters, consistent with observations that mortalities tend to occur in summer when oysters have low energetic resources, energy demand and high reproductive effort [66,67]. At all locations, there were many rises and falls in water temperature over time, not only towards the end of the subclinical infection period, so perhaps the cumulative effect of these is important. The relatively rapid changes in temperature at higher temperatures may have immunologically compromised oysters through stress and tipped the host–pathogen balance in favour of OsHV-1 or cofactors such as bacteria [68]. It is possible that this triggered progression from subclinical infection into the incubation period. 

Sea surface temperature patterns at the entrance to each estuary compared to long-term records did not show any consistent anomaly between the four index cases. At the Georges River, sea surface temperature was close to the long-term average for the entire subclinical period. In contrast, at the Hawkesbury River, it was 1 °C to 2 °C below the long-term average while in both index cases in TAS, it was up to 2.5 °C higher. For the Lower Pittwater TAS index case, the water temperatures around Storm Bay then were among the highest ever recorded due to the unusual southern penetration of the East Australian current. It is reasonable to assume that estuarine temperatures followed the same trends over time because they were moderately correlated to sea surface temperatures, but they would have been several degrees warmer than sea surface temperature (see Table 3 and Figure 3 for comparisons). Despite Lower Pittwater TAS having record high sea surface temperatures, the water temperatures there were about 3 °C to 5 °C below those of the Hawkesbury River and Georges River, respectively (Figure 6, Table 2 and Table 3), which correlates with their respective latitudes. 

While the atmospheric heatwave in the Sydney region in January 2013 could have been a factor in the Hawkesbury River index case, extreme air temperatures were not a feature in the other outbreaks. It has been suggested that the marine heat wave of 2015/2016 explains the first outbreak of POMS in Tasmania [69], but a marine heat wave was not underway during the other three index cases; their water temperatures were not unusual based on long-term records. Nevertheless, a marine heat wave may have enabled the first outbreak in Tasmania in the sense that it might not have been possible in a cooler year because the threshold temperature for mortality might not have been reached.

Temperature could influence POMS in a number of different ways including via effects on filter feeding because the virus is attached to particles in the water column upon which oysters feed [34,35,70]. However, *C. gigas* feeds between 3 °C and 35 °C with an optimum of 11–34 °C [71], a wider temperature range than is permissive for POMS; therefore, temperature must act primarily via another mechanism. Effects on the viral replication rate [38], host metabolism and immunity [72], the oyster microbiome, particularly *Vibrio* sp. [73,74,75], and the presence or absence of particulate transmission vectors and competing filter feeders in the water column [70,76] could all be involved. 

### 4.2. Salinity

*C. gigas* has a wide salinity tolerance (10–42 ppt) [71]. In OsHV-1 challenge experiments, 10 ppt was protective against mortality compared to 15, 25 and 35 ppt, and there was progressively higher mortality with increasing salinity across that range [77]. The effect was attributed to reduced infectivity of the virus at low salinity as well as effects on bacterial communities that contribute to pathogenesis [77]. 

Salinity in the Georges River NSW at the onset of mass mortality was 30.9 ppt [16], following 24 mm of rain during the incubation period (Table 3). Prior monitoring of salinity in the lower Georges River/Woolooware Bay conducted over a full year revealed levels of 26 ppt to 35 ppt [78], although much longer-term monitoring revealed levels generally lower than this range and less than the oceanic boundary of 35.6 ppt [79]. Continuous monitoring by the authors on two oyster leases in Woolooware Bay Georges River between October 2011 and May 2012 produced a range of 18 ppt to 36 ppt and a median of 31 ppt suggesting that Woolooware Bay is more typical of the lower estuary than the adjoining and typically oceanic Botany Bay, in agreement with Loveless [80]. During the long subclinical period in the Hawkesbury River, salinity reflected low rainfall and freshwater inflows and was in the range 32 ppt to 36 ppt. Although salinity data were not available for Pittwater Tasmania for the period of the outbreak, monitoring from 1991 to 1994 revealed values generally over 32 ppt and sometimes greater than marine conditions (hypersaline) [44]. Rainfall had been very low in the region, less than 5mm fell during the incubation period and less than 100 mm during the entire subclinical infection period (Table 3 and Table 4). It can be assumed that the Pittwater estuary in TAS had oceanic salinity. Salinities in Georges Bay TAS were over 32 ppt consistent with observations made over one year in 1993–1994 [44]. 

It is reasonable to conclude that all four index cases occurred with salinity levels above the protective level of 10 ppt and most likely above 30 ppt, that is, at levels conducive to mass mortality.

### 4.3. Particulate Vectors, Tides and Algae

Transmission of OsHV-1 to *C. gigas* involves the attachment of virions to particles, that is, a biotic or abiotic planktonic carrier [35,70]. POMS can be prevented by keeping oysters in filtered seawater [81,82] and the presence of other invertebrate filter feeders can mitigate the impact of OsHV-1 on *C. gigas* [76]. Particles suspended from sediments by tidal currents and algae (represented in chlorophyll-a measurements), could be important in OsHV-1 transmission. 

While estuary chlorophyll-a levels were low throughout most of the subclinical infection period and when mortalities began in both the Georges River NSW and Hawkesbury River NSW index cases, the levels had doubled during both incubation periods. An algal bloom in the vicinity of the oyster leases was noticed by farmers around the time of the first mortalities in the Georges River [16]. Chlorophyll-a data were not available for either index case in TAS, but historical records from 1991 to 1994, a period long enough to encompass a range of typical weather events showed chlorophyll-a levels for Pittwater TAS of 1 to 8 µg/L and for Georges Bay of 1–4 μg/L with occasional spikes [44]. These values were within the range observed in the two NSW estuaries. There had been a long period of low rainfall in TAS prior to both index cases, therefore nutrient input to estuaries from freshwater runoff and the probability of algal bloom would both have been low.

Spring tides have greater potential to cause resuspension of particles than neap tides and they could facilitate transmission of OsHV-1 through particle attachment. Flow velocity in the mid-Georges River estuary during a spring tide was 0–0.4 m/s compared to 0.2 m/s in a neap tide [79]. However, tidal influences at the onset of mortality were inconsistent between the four index cases being either spring, neap or approaching neap. 

Beyond their potential role in viral attachment, some algae can directly impact oyster health [47]. Toxic microalgae species, in particular *Alexandrium minutum* and *A. catenella*, have been reported during OsHV-1-related mortalities in France [83]. Since 2005, there has been an increase in the frequency of blooms of *Alexandrium* in south-eastern Australian coastal waters [84]. *Alexandrium* species in the downstream areas of the Hawkesbury River tend to be present all year at extremely low counts and the levels tend to peak between September and November every year [84]. *A. catenella* was present in the Hawkesbury River during the subclinical infection period, in October and December 2012 (50–300 cells/L). However, *Alexandrium* species were not detected at the time of mass mortality in the Hawkesbury River or at any relevant time in Lower Pittwater and only low levels were found in Georges Bay. *A. catenella* was detected during the subclinical infection period in the Georges River, at low levels in September and October 2010 with levels increasing by several orders of magnitude during the incubation period and as previously reported reached 20,000 cells/L at the onset of mortality [16]. Even higher counts were observed during oyster mortalities in France (10^5^ to 10^6^ cells/L) [85,86]. It is possible that phytoplankton blooms in the Georges River facilitated virus transmission or negatively affected oyster health in some way to induce mortality. Indeed, the histological changes associated with the presence of toxin-producing *Alexandrium* spp., which included haemocyte accumulation in the connective tissue surrounding the gut and digestive tubules [87] were also observed in the Georges River index case oysters [16]. Substantial counts of the *Pseudo-nitschia seriata* group were present in the subclinical infection period in both index cases in Tasmania while the *Pseudo-nitschia delicatissima* group was present at a high level (>50,000 cells/L) in Georges Bay. Although the observations were infrequent, there is potential consistency between the index cases that merits further investigation of harmful algae in the emergence and pathogenesis of POMS.

### 4.4. Environmental Contamination and Toxins

Beyond the effect on salinity, freshwater inflows from a catchment may be accompanied by ingress of organic matter, nutrients and pollutants to an estuary. Following 24 mm of rainfall during the incubation period, discoloured water was seen near the affected oyster leases in the Georges River NSW [16]. These lie adjacent to a creek with a golf course and urban area in its immediate catchment. However, significant levels of organic compounds and metals were not detected [16]. The Hawkesbury River NSW and Lower Pittwater TAS estuaries both have large urban catchment areas, and so would also be subject to a wide range of potential toxicological influences, especially after a rain event, but rainfall at both locations was below long-term averages with no unusual rainfall events during the subclinical and incubation periods. It seems unlikely that anthropogenic toxins played a role in the index cases.

### 4.5. Limitations

The environmental conditions evaluated in this study were limited by the availability of data collected from oyster lease areas as well as by the publicly available meteorological and oceanic data, only some of which were available from high-frequency measurements or over decades. While constrained, it was possible to evaluate temperature, salinity, rainfall, chlorophyll-a and tides and to a lesser extent harmful algae, which enabled assessment of some of the factors that could influence pathogenesis. There are many other environmental factors for which there were no data. For example, chemicals and pollutants such as endocrine disruptors, pesticides and metals are relevant but direct data were obtained from only one of the index cases and we relied on inference from rainfall data to assess the risk of contamination. It was not possible to properly assess the role of environmental vectors and mitigating agents such as other filter feeders due to a lack of ecological data from the estuaries. Finally, there may have been different environmental risk factors for each index case, possibly acting through a common mechanism, such as immune suppression. A larger study with many more index cases would be required to identify such factors, for example, through logistic regression analysis.

### 4.6. Inferences on the Origin of the Virus for Index Cases

OsHV-1 has been spread within and between countries in Europe by unrestricted anthropogenic movements of oysters [11,13]. These include transfers of spat from commercial sources mainly in France [11] and also between farms. While numerous aquatic animal pathogens have been introduced into Australian waters with imports of live animals or animal products (for examples see [88,89,90]), there have been no imports of live oysters for farming purposes in Australia for many decades. Furthermore, biosecurity regulations imposed in 2010 make it very unlikely that OsHV-1 was spread from hatcheries or among oyster farmers in Australia. 

Commercial farming of *C. gigas* began in Tasmania in the 1960’s [91], in the Georges River NSW in 2004 and in the Hawkesbury River NSW in 2005 [92], all based on *C. gigas* imported into Australia in 1947 from Japan [93]. OsHV-1 does not cause mass mortality in Japan [63], but it is possible that non-pathogenic variants of OsHV-1 were introduced from Japan with the original oyster imports and have persisted. 

Mass mortality in *C. gigas* was unknown in Australia prior to 2010 and while tests for OsHV-1 were not performed before then, it seems unlikely that pathogenic variants of OsHV-1 existed in farmed *C. gigas* populations due to the absence of disease. Intensive active surveillance was conducted in 2011 in all Pacific oyster-growing regions in NSW, TAS and South Australia [51,94]. OsHV-1 was not detected beyond the known Georges River NSW index case. Based on this survey, the history of freedom from disease and field epidemiology, an external source of OsHV-1 has been proposed [35]. This was reinforced when sequencing data revealed the unique identities of the Australian OsHV-1 and the fact that they differed between the three index cases reported up until 2022 [21]. That is, the virus was not translocated between the Georges River index case and the two that followed. 

In the absence of consistent temperature patterns and anomalies, it can be concluded that the thermal environment at each location was merely suitable for viral transmission and pathogenesis. Among the other environmental parameters described here, we did not find a consistent factor for the index cases apart from low rainfall. Three of four index cases followed long periods of unusually low rainfall and in the fourth, there was low rainfall during most of the subclinical infection period. Considering also the season, each index case arose after an unusually dry spring, and three of them after a dry winter/spring. While harmful algae were present and may have contributed in some way, environmental contamination of estuaries from surface run-off was unlikely due to the low rainfall in three index cases. The rain event during the Georges River index case incubation period was not accompanied by demonstrable contamination [16].

It is unclear how low rainfall could enable the emergence of the virus in the local environment beyond indirect influences on estuarine ecology through reduced nutrient inflows or stable salinity, but further investigation is warranted. Overall, the findings may be consistent with the recent introduction of the pathogen rather than its emergence from a local reservoir caused by a change in the local environment. However, only a small number of environmental factors were studied, critical factors may not have been discovered and the effects of rainfall are not understood. For this reason, and to explain the fact that the variants of the virus responsible for these outbreaks were different, local emergence cannot be excluded and translocation of OsHV-1 from three different sources beyond the affected estuaries must also be considered. Due to the lack of evidence for anthropogenic spread or biosecurity failure, translocation could be a result of natural transport by oceanic currents possibly over long distances. New emergence events or introductions are likely over time but will be difficult to predict. 

It is necessary to develop comprehensive protocols for investigation and data acquisition during future index cases to properly understand the source of OsHV-1. Temperature appears to be a proxy for a range of undetermined factors. In Tasmania, recent data modelling suggested that a 21-day average water temperature of 23.7 °C resulted in a 70% likelihood of >15% mortality [95], but the causes of mortality were not differentiated. Further research is being undertaken on temperature profiles in recurrent seasonal outbreaks of POMS in the Georges River and Hawkesbury River estuaries, and the risks presented by local temperature profiles in other estuaries are also being evaluated. 

## 5. Conclusions

While temperature is an important factor in seasonally recurrent, endemic POMS, there was no evidence of consistent sea surface or atmospheric temperature patterns in the year prior to each index case that could explain OsHV-1 emergence. However, the rates of increase in water temperature during temperature fluctuations in spring and summer were greater at higher temperatures at each location, raising the possibility that cumulative thermal change stress triggered progression from subclinical infection into the incubation period in oysters that were otherwise adapted to their different latitudinal locations. There was a wide thermal range for the onset of mass mortality among the four index case locations, with average daily estuarine water temperatures being 20–25 °C, and with similar inconsistency in sea surface temperatures and occurrence of marine or atmospheric heat wave conditions. This suggests that the water temperatures at each location had merely been permissive for the emergence of OsHV-1 and the eventual onset of POMS. All index cases occurred with relatively high salinity levels, well above the protective level of 10 ppt. Tidal influences at the onset of mortality were inconsistent between the four index cases, and being a regular recurrent feature would normalise between sites over time. Chlorophyll-a levels were unremarkable, and while observations of harmful algae were infrequent, they were detected at relevant times in the genesis of all four index cases and this merits further investigation. Negative anthropogenic contaminant test results for one index case and unusually low rainfall and hence runoff into estuaries for the other three index cases make contaminant involvement unlikely. The lack of an obvious or interpretable change in the local estuarine environment that could explain emergence from a local reservoir suggests the recent introduction of the pathogen to each estuary. However, because only a small number of environmental factors were studied, local emergence cannot be excluded. New emergence events are likely to occur over time but will be difficult to predict. 

The occurrence of index cases during the warmer months following long periods of unusually dry weather identified temperature and rainfall as important factors, but they are proxies for a range of undetermined factors. To identify these factors, it will be necessary to develop comprehensive protocols for data acquisition during future index cases.

## Figures and Tables

**Table 1:** Dates for each index case, 10-day incubation period and subclinical infection period. The intervals for analysis begin and end at 12:00 midday on each date.

Location and Coordinates (Decimal Degrees)	Period	Interval for Analysis
Georges River estuary, Woolooware Bay, NSW −34.008950, 151.138058	Index case	23/11/2010 12:00 to 24/11/2010 12:00
−34.008950, 151.138058	Incubation period	13/11/2010 12:00 to 23/11/2010 12:00
	Subclinical infection	23/07/2010 12:00 to 23/11/2010 12:00
Hawkesbury River estuary, Mullet Creek, NSW	Index case	20/01/2013 12:00 to 21/01/2013 12:00
−33.533490, 151.220970	Incubation period	10/01/2013 12:00 to 20/01/2013 12:00
	Subclinical infection	20/09/2012 12:00 to 20/01/2013 12:00
Lower Pittwater estuary, TAS	Index case	26/01/2016 12:00 to 27/01/2016 12:00
−42.812896, 147.540608	Incubation period	16/01/2016 12:00 to 26/01/2016 12:00
	Subclinical infection	26/09/2015 12:00 to 26/01/2016 12:00
Georges Bay, TAS	Index case	03/02/2024 12:00 to 04/02/2024 12:00
−41.312674, 148.288326	Incubation period	24/01/2024 12:00 to 03/02/2024 12:00
	Subclinical infection	03/10/2023 12:00 to 03/02/2024 12:00

**Table 2:** Index case period conditions. Values are the daily mean, standard deviation, minima and maxima calculated from data recorded at 15 min intervals and total rainfall for 24 h prior to midday on the date of onset of mass mortality. Data sources are described in the Materials and Methods; exceptions are provided in the footnotes.

Variable	Georges River, NSW 16 November 2010				Hawkesbury River, NSW 21 January 2013					Lower Pittwater, TAS 27 January 2016				Georges Bay, TAS 4 February 2024		
	Mean ± s.d.	Min	Max		Mean ± s.d.	Min	Max			Mean ± s.d.	Min	Max		Mean ± s.d.	Min	Max
Air temp (°C)	22.2 ± 2.3	18.1	26.1		23.4 ± 2.1	21.3	28.5			16.8 ± 2.2	13.3	20.7		21.3 ± 3.7	17.3	27.9
Sea surface temp (°C)	20.1 ± 0.1	20.0	20.1		22.4 ± 0.1	22.3	22.4			18.5 ± 0.1	18.4	18.6		19.0 ± 0.1	18.9	19.0
Estuary water temp (°C)	22.7 ± 0.4	22.0	23.5		24.1 ± 0.3	23.7	24.5			-	-	-		-	-	-
Oyster-lease water temp (°C)	-	-	-		25.1 ± 0.3	24.5	25.4			20.1 ± 0.4	19.5	21.0		19.7 ± 0.1	19.6	20.0
Total rainfall (mm)	0	-	-		3	-	-			0.7	-	-		0	-	-
Estuary salinity (ppt)	30.9 ^1^	-	-		35.3 ± 0.3	34.7	35.6			-	-	-		34.0 ± 0.7	32.1	35.0
Estuary chlorophyll-a (ug/L)	4.7 ± 1.0	2.7	6.0		2.9 ± 0.8	1.5	6.2			-	-	-		-	-	-
Water level/tides (m)	Spring	0.3	1.8		Neap	0.6	1.4			Approaching neap	0.2	1.1		Neap	0.3 ^2^	1.0 ^2^

^1^ Data from [16], ^2^ St Helens TAS predicted tide levels.

**Table 3:** Incubation period conditions. Values are the mean, standard deviation, minima and maxima calculated from data recorded at 15 min intervals and total rainfall over 10 days prior to the day of onset of mass mortality. Data sources are described in the Materials and Methods; exceptions are provided in the footnotes.

Variable	Georges River, NSW				Hawkesbury River, NSW					Lower Pittwater, TAS				Georges Bay, TAS		
	Mean ± s.d.	Min	Max		Mean ± s.d.	Min	Max			Mean ± s.d.	Min	Max		Mean ± s.d.	Min	Max
Air temperature (°C)	20.7 ± 3.3	13.9	32.2		25.2 ± 5.9	16.4	47.8			18.0 ± 2.9	13.0	26.2		19.0 ± 4.0	10.6	27.7
Sea surface temperature (°C)	20.2 ± 0.2	19.8	20.6		21.9 ± 0.4	21.2	22.4			17.8 ± 0.4	17.2	18.6		18.9 ± 0.2	18.6	19.2
Estuary water temperature (°C)	23.0 ± 0.7	21.2	25.0		23.9 ± 0.6	22.5	26.0			-	-	-		-	-	-
Oyster-lease water temp (°C)	-	-	-		25.8 ± 0.9	23.7	28.1			21.2 ± 1.2	19.5	25.5		19.5 ± 0.5	18.3	22.0
Total rainfall (mm)	23.9	-	-		16.0	-	-			4.3	-	-		0.3	-	-
Estuary salinity (ppt)	-	-	-		34.9 ± 0.4	34.0	35.6			-	-	-		34.4 ± 0.7	30.0	35.1
Estuary chlorophyll-a (ug/L)	4.9 ± 1.2	2.1	11.8		2.3 ± 0.7	0.6	7.3			-	-	-		-	-	-
Water level/tides (m)	Figure 3	0.3	1.8		Figure 3	0.1	2.0			Figure 3	0.3	1.6		Figure 3	0.1 ^1^	1.2

^1^ St Helens TAS predicted tide levels.

**Table 4:** The duration of exposure of oysters to water temperatures exceeding thresholds for periods of at least 3 h during the 10-day incubation period at each location. Data were from observations every 15 min.

Threshold Temperature (°C)	Duration (h)	Mean Temp ^1^ (°C)
Georges River, NSW		
21	240	23.0
22	226	22.7
23	74	23.7
24	20	24.3
Hawkesbury River, NSW		
22	240	23.9
23	233	23.7
24	69	24.5
25	8	25.4
Lower Pittwater, TAS		
19	240	21.2
20	196	21.3
21	96	22.3
22	42	23.1
23	17	24.0
Georges Bay, TAS		
18	240	19.5
19	205	19.5
20	24	20.4

^1^ mean water temperature attained during periods when the threshold was exceeded.

**Table 5:** Subclinical infection period conditions. Values are the mean, standard deviation, minimum and maximum values of average daily data and total rainfall over 4 months prior to the date of onset of mass mortality. Data sources are described in the Materials and Methods; exceptions are provided in the footnotes.

Variable	Georges River, NSW				Hawkesbury River, NSW				Lower Pittwater, TAS				Georges Bay, TAS ^1^		
	Mean ± s.d.	Min	Max		Mean ± s.d.	Min	Max		Mean ± s.d.	Min	Max		Mean ± s.d.	Min	Max
Air temperature (°C)	16.6 ± 3.2	11.3	25.6		21.7 ± 3.5	12.4	31.5		15.6 ± 3.4	9.4	26.2		15.6 ± 2.7	9.3	20.7
Sea surface temperature(°C)	18.4 ± 1.1	16.7	20.8		19.7 ± 1.4	17.2	22.4		14.5 ± 1.9	11.2	18.6		16.7 ± 2.1	13.5	19.3
Estuary water temperature (°C)	17.5 ± 2.9	13.8	24.1		21.1 ± 2.1	17.6	25.0		-	-	-		-	-	-
Oyster-lease water temp (°C)	-	-	-		22.6 ± 2.5	18.4	27.6		17.9 ± 2.4	13.1	22.2		17.8 ± 2.1	13.6	21.2
Rainfall (mm)	2.9 ± 7.3	0	42		1.4 ± 3.8	0	30		0.7 ± 1.9	0	14		1.5 ± 4.1	0	33.5
Total rainfall (mm)	357	-	-		174	-	-		82	-	-		183	-	-
Estuary salinity (ppt)	-	-	-		35.0 ± 0.6	33.6	36.3		-	-	-		33.4 ± 2.9	16.1.	35.3
Estuary chlorophyll-a (ug/L)	3.8 ± 1.0	2.1	7.0		2.1 ± 0.7	0.6	5.6		-	-	-		-	-	-
Daily tidal range (m)	1.2 ± 0.3	0.8	1.9		1.3 ± 0.3	0.8	1.9		0.9 ± 0.2	0.5	1.4		0.8 ± 0.2	0.4	1.2

^1^ Tide levels recorded at Spring Bay TAS.

**Table 6:** The duration of exposure of oysters to mean daily water temperatures exceeding thresholds for periods of at least three days, and rates of increase in water temperature during the 4-month subclinical infection period at each location. Data were from daily average observations.

Threshold Temperature (°C)	Duration (Days)	Mean Temp ^1^ (°C)	Mean rate of Increase (°C/Day)
Georges River NSW			
15	84	18.9	0.1
16	70	18.1	0.2
17	63	19.9	0.1
18	57	19.4	0.2
19	44	20.9	0.4
20	18	21.6	0.5
21	14	22.7	0.7
22	12	22.9	0.7
23	4	23.7	0.7
Hawkesbury River NSW			
17	123	21.1	-
18	112	19.5	0.2
19	96	21.9	0.1
20	81	21.3	0.1
21	62	22.2	0.1
22	50	22.8	0.2
23	24	23.7	0.4
24	9	24.4	0.4
Lower Pittwater TAS			
15	107	17.6	0.2
16	90	17.7	0.5
17	63	18.7	0.4
18	52	19.6	0.3
19	41	20.7	0.4
20	31	21.1	0.6
21	15	21.5	0.5
Georges Bay TAS			
15	100	17.0	0.1
16	91	18.9	0.1
17	85	18.7	0.1
18	66	19.0	0.2
19	43	19.6	0.3
20	15	20.5	0.1

^1^ mean water temperature attained during periods when the threshold was exceeded.

## Data Availability

Most of the data presented in this study are available from public sources described in the materials and methods; aggregated data for each location are available from the corresponding author upon request.
